# Headway and the remaining hurdles of mesenchymal stem cells therapy for bronchopulmonary dysplasia

**DOI:** 10.1111/crj.13540

**Published:** 2022-09-02

**Authors:** Eireen Tang, Mariam Zaidi, Wen‐Huey Lim, Vijayendran Govindasamy, Kong‐Yong Then, Khong‐Lek Then, Anjan Kumar Das, Soon‐Keng Cheong

**Affiliations:** ^1^ CryoCord Sdn Bhd, Bio‐X Centre Cyberjaya Malaysia; ^2^ Brighton Healthcare (Bio‐X Healthcare Sdn Bhd), Bio‐X Centre Cyberjaya Malaysia; ^3^ Department of Surgery IQ City Medical College Durgapur India; ^4^ Faculty of Medicine & Health Sciences, Universiti Tunku Abdul Rahman (UTAR) Kajang Malaysia

**Keywords:** chronic lung department, extremely low gestational age newborns (ELGANs), mesenchymal stem cells, PNEUMOSTEM, preterm infants, respiratory diseases

## Abstract

**Objective:**

Preterm infants are at a high risk of developing BPD. Although progression in neonatal care has improved, BPD still causes significant morbidity and mortality, which can be attributed to the limited therapeutic choices for BPD. This review discusses the potential of MSC in treating BPD as well as their hurdles and possible solutions.

**Data Sources:**

The search for data was not limited to any sites but was mostly performed on all clinical trials available in ClinicalTrials.gov as well as on PubMed by applying the following keywords: lung injury, preterm, inflammation, neonatal, bronchopulmonary dysplasia and mesenchymal stem cells.

**Study Selections:**

The articles chosen for this review were collectively determined to be relevant and appropriate in discussing MSC not only as a potential treatment strategy for curbing the incidence of BPD but also including insights on problems regarding MSC treatment for BPD.

**Results:**

Clinical trials regarding the use of MSC for BPD had good results but also illustrated insights on problems to be addressed in the future regarding the treatment strategy. Despite that, the clinical trials had mostly favourable reviews.

**Conclusion:**

With BPD existing as a constant threat and there being no permanent solutions, the idea of regenerative medicine such as MSC may prove to be a breakthrough strategy when it comes to treating BPD. The success in clinical trials led to the formulation of prospective MSC‐derived products such as PNEUMOSTEM®, and there is the possibility of a stem cell medication and permanent treatment for BPD in the near future.

## Graphical abstract

The attention focused on mesenchymal stem cells for the treatment of bronchopulmonary dysplasia proved to be a potential solution to eradicate its incidence permanently. The implementation of such a treatment, however, has some ways to go, considering it brings about its own challenges that have to be addressed.

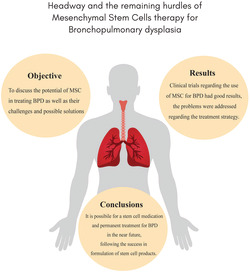

**Abbreviations**
aCGH: array‐comparative genomic hybridization
BASCs: bronchoalveolar stem cells
BMSCs: bone marrow stem cells
BPD: bronchopulmonary dysplasia
CNV: copy number variants
COPD: chronic obstructive pulmonary disorder
ELGAN: extremely low gestational age newborn
EMA: European Medicines Agency
FDA: Food and Drug Administration
GA: gestational age
GVDH: graft‐versus‐host disease
hAEC: human amnion epithelial cells
HGP: hepatocyte growth factor
HO: heme oxygenase
hUC: human umbilical cord
hUCB: human umbilical cord blood
IDO: indoleamine 2,3‐dioxygenase
IL: interleukin
IUGR: intrauterine growth restriction
KLF: Krueppel‐like factor
Miro: mitochondrial rho GTPase
MSC: mesenchymal stem cells
MSC‐CM: mesenchymal stem cell conditioned media
MVB: multivesicular body
Myo: myosin
NF‐κB: nuclear factor kappa‐light‐chain‐enhancer of activated B cells
PDA: patent ductus arteriosus
PIGF: placental growth factor
RDS: respiratory distress syndrome
ROS: reactive oxygen species
sFlt: tyrosine kinase
TGF: transforming growth factor
TNF: tumour necrosis factor
TNT: tunnelling nanotubes
TRAK: trafficking kinesin‐binding protein
Treg: regulatory T cells
TSG: TNF‐stimulated gene
VEGF: vascular endothelial growth factor

## 1 INTRODUCTION

Despite a considerable number (241) of in‐human clinical trials registered in ClinicalTrials.gov and the progression in neonatal medicine witnessed in the last five decades, the problem of lack of good therapeutic alternatives for bronchopulmonary dysplasia (BPD) continues to exist. Treatment strategies (Table 1) ranging from medications (antibiotics and diuretics) to steroid treatments all proved incapable of curing BPD and instead only aimed to aid the breathing of afflicted individuals.^1^, ^2^, ^3^, ^4^, ^5^, ^6^, ^7^, ^8^ As the occurrence of BPD correlates with prematurity and extremely low gestational age newborns (ELGANs), with approximately every 50 000 ELGANs born, the numbers that develop BPD exceeds that of 18 000.^9^ The past few decades have witnessed a plateau of about 40% in the incidence of BPD within infants of less than or equal to 28 weeks gestational age (GA).^10^, ^11^, ^12^ However, based on the data acquired from cohort studies including Canadian, Korean, Swiss Neonatal Networks, Vermont‐Oxford Network, and research in China, Taiwan, and India, the prevalence of BPD lies between 11% and 50%.^9^ This marks out BPD as a constant and persistent threat in every community, desperately calling for the need of a new, innovative and comprehensive treatment strategy.

**TABLE 1:** Conventional prevention/treatment strategies against BPD

Interventions	Clinical response	Side effects	References
Bronchodilators	Reduce pulmonary resistance	Tachycardia, hypokalaemia, arrhythmias, and hyperglycaemia	^1^, ^2^, ^3^
Caffeine	Anti‐inflammatory and reduce dependency on mechanical ventilation	Reduce weight gain	^1^, ^2^, ^3^, ^4^
Diuretics	Improve pulmonary oedema and decrease pulmonary vascular resistance	Ototoxicity, electrolyte disturbances, and azotaemia	^1^, ^2^, ^3^, ^4^
Fluid restriction	Improve pulmonary oedema and reduction in BPD incidence/severity	May interfere with the recommended nutrient intake	^5^
Steroids (systemic, inhaled)	Anti‐inflammatory and improve oxygenation	Hyperglycaemia, gastrointestinal perforation, and hypertension	^1^, ^3^, ^6^, ^7^
Inhaled nitric oxide (NO)	Ongoing evaluation	No side effects reported, ongoing evaluation	^1^
Inositol	Improve pulmonary function and reduce BPD incidence/mortality	No side effects reported	^1^, ^2^, ^3^
Macrolide antibiotics	Anti‐inflammatory and reduce BPD incidence and mortality rate	No side effects reported	^4^
Nutrition supplementation	Prevent growth failure of infants	No side effects reported	^5^, ^6^
Supplemental oxygen/ventilator support	Improve oxygenation	Require hospitalization/home oxygen therapy	^1^
Surfactant	Decrease mortality and severity of respiratory distress syndrome	No side effects reported	^2^, ^4^, ^6^
Vitamin A	Improve lung development and growth	High cost and low availability	^1^, ^2^, ^3^, ^4^, ^5^, ^6^, ^8^

A condition often unspoken about in the community, BPD remains listed as one of the frequent players in the chronic lung disease department in children under 5, third only to asthma and cystic fibrosis. It is one of the most prevalent and severe respiratory complications resulting from extreme prematurity. Described as a chronic lung disease which leads to the requirement of mechanical ventilation and oxygen therapy during infancy and long‐term respiratory effects in adults, BPD causes a depletion in alveolarization, vascular growth and lung function, resulting in perinatal inflammation and oxidative stress, all of which consequently disrupt lung development.^13^ Its occurrence is most observed in preterm infants who are born at 24–26 weeks of gestation with a birth weight of less than 1000 g, forcing the infants to require and rely on extensive oxygen therapy and ventilation support for survival.^14^ Children with BPD are also more prone to developing cognitive and motor impairment, speech, language, visual and auditory disorders, and behavioural problems.

## 2 PATHOPHYSIOLOGY OF BPD

BPD possesses a multifactorial aetiology comprising pre‐ and post‐natal agents responsible for detrimental alveolar growth with preterm birth being the governing factor. This includes prenatal inflammation and infection, oxygen toxicity combined with declining host antioxidant defences, mechanical ventilation, patent ductus arteriosus and postnatal infection with the additive influence of genetic factors as major causative agents of BPD.^15^ Studies have also characterized preeclampsia as another causative agent.^16^, ^17^ Disrupted signalling of vascular endothelial growth factor (VEGF) and impaired angiogenesis in preeclampsia are additional factors associated with BPD development.^18^, ^19^, ^20^, ^21^, ^22^

### 2.1 Lung development

Lung development begins between 3 and 6 weeks of gestation where efficient respiration depends on the structure of peripheral lung saccules and alveoli. At 28–40 weeks of gestation, the peripheral lung saccules undergo septation wherein the airspaces are divided thus elevating the number of alveoli, consequently magnifying the surface area for gas exchange. The peripheral lung epithelial cells develop into alveolar type 1 and type 2 cells out of which, alveolar type 2 processes pulmonary surfactant. Therefore, premature infants at 22–23 weeks GA start ventilation at the canalicular–saccular stage of lung development which is prior to the completion of morphogenesis and alveolar differentiation.^9^

Premature birth within the early stages of saccular phase is accompanied by impaired lung development and septation. Subsequently, infection, ventilation and supplemental oxygen provokes the pro‐inflammatory pulmonary response which in turn impacts healthy lung development leading to a reduction in the number of alveoli and diminished alveolar anatomy leading to a decrease in gas exchange surface area.^21^ Furthermore, lung growth during the alveolar phase fails to generate an updated development leading to detrimental lung functionality persevering into adulthood.^23^ A deviated pulmonary vascular growth due to impaired air‐conducting segments of the lung were seen within patients with severe BPD resulting in a situation of dual limitation of gas transport caused by restricted lung surface area and decreased capillary network.^21^

### 2.2 Pulmonary surfactant

An effective structure, function and metabolism of surfactant within the alveolus depends on a combination of phospholipids such as phosphatidylcholine and proteins including SP‐A, SP‐B, SP‐C and SP‐D. The formation of these surfactant lipids and proteins is dependent on differentiating alveolus type 2 cells occurring during late gestation. However, when the body is low on pulmonary surfactant due to unfinished differentiation, an illness known as respiratory distress syndrome (RDS) develops amongst infants.^24^ Although administering exogenous pulmonary surfactant within infants lowers oxygen requirement and mechanical ventilation within preterm infants, it increases their risk of acquiring BPD.^9^

### 2.3 Patent ductus arteriosus

Patent ductus arteriosus (PDA) is linked with deleterious respiratory outcomes and a study by Brown^25^ hinted at the correlation between BPD and PDA, specifically amidst infants with extremely low birth weight. Another study established the increasing risk of BPD in premature infants weighing between 500 and 1000 g upon the synchroneity of PDA and infection.^26^ Rojas et al.^27^ determined that significant causative agents of BPD included PDA, sepsis and low birth weight where the inclusion criteria involved (1) premature infants weighing between 500 and 1000 g; (2) undergoing mechanical ventilation; (3) requiring less than 3 days of fraction of inspired oxygen (more than 25% within the initial 5 days of birth); and (4) having survived at least 28 days who, due to the co‐occurrence of PDA and sepsis, have increasing odds for BPD compared with infants devoid of these symptoms.

### 2.4 Mechanical ventilation

The implementation of positive pressure ventilation for preterm infants could lead to BPD due to mechanical lung overdistension and alveolar stretch. This happens as excess volume and pressure can cause lung damage by overinflating the alveoli, causing cellular injury, inflammation and reactive oxygen species (ROS) generation, which contributes to the amplification of injury caused by prenatal inflammation.^28^

### 2.5 Oxygen toxicity

Oxygen toxicity is another risk factor of BPD as it has been observed in animal models that supraphysiologic oxygen solely compromises alveolar development and pulmonary vascular remodelling. These models have showcased long‐term modifications of the lung upon exposure to high concentrations of oxygen.^29^, ^30^ Clinical details suggest that even a slight exposure of supraphysiologic oxygen whilst resuscitating enhances BPD risk and development.^31^ Moreover, preterm infants are prone to experiencing oxidative stress due to underdeveloped antioxidant defences, vulnerability to infection and subjection to free iron.^32^

### 2.6 Genetic predisposition

Having recently emerged as a possible agent in BPD development, genetic predisposition is a potential causative factor of BPD as reported by Bhandari et al.^33^ wherein 450 pairs of twins were assessed where hereditary and shared environmental agents account for 65% of variances in BPD sensitivity.^28^ Numerous other studies also address the linkage between genetic polymorphisms and BPD development.^34^, ^35^, ^36^, ^37^

### 2.7 BPD phenotype

**FIGURE 1:**
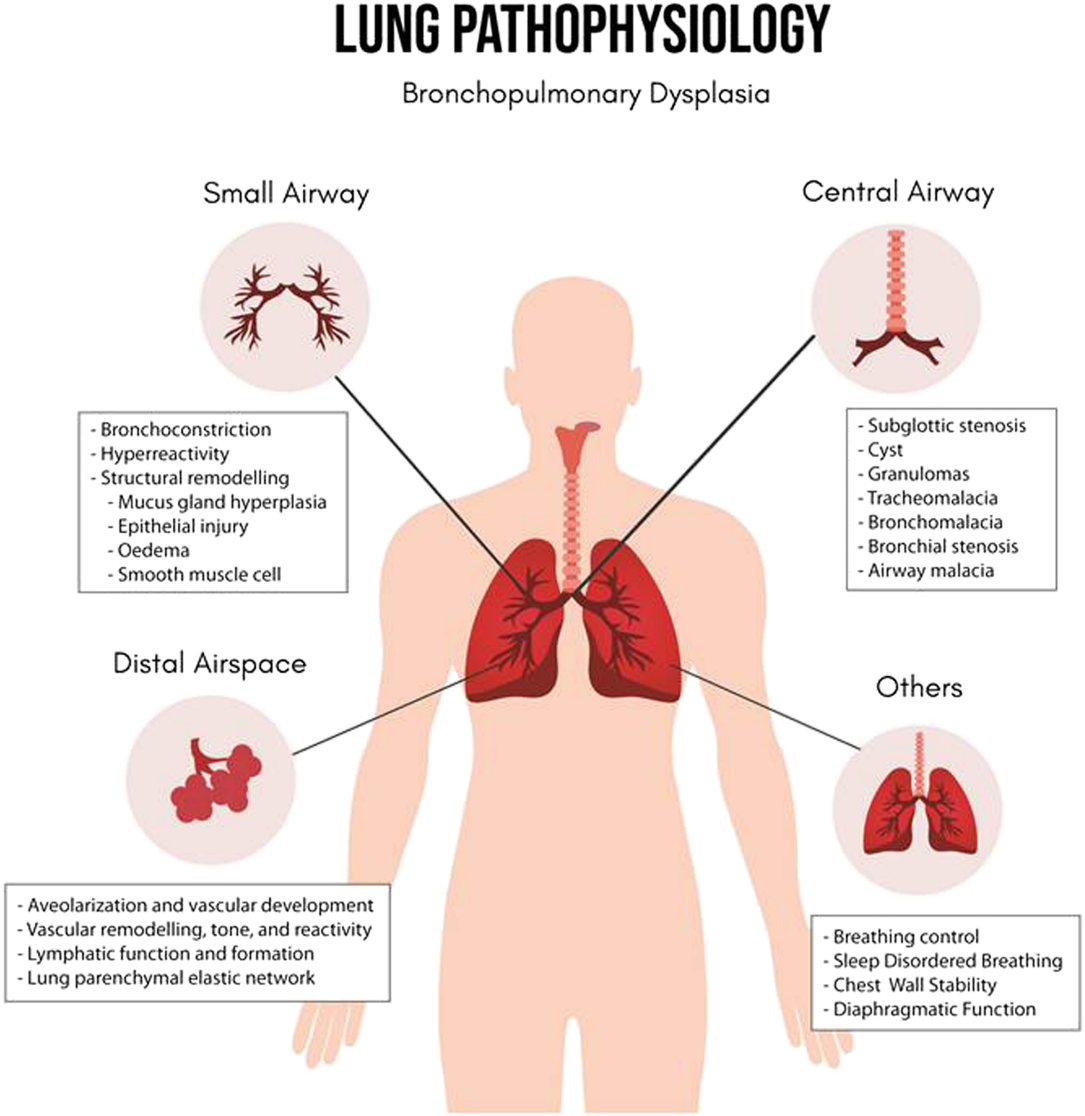
Lung pathophysiology affected by BPD

Preterm infants who at birth are small for gestational age or possess intrauterine growth restriction (IUGR) have a higher risk of acquiring pulmonary damage.^5^, ^38^ For instance, researchers have determined a twofold elevated risk of BPD (28% vs. 14%) and neonatal mortality (23% vs. 11%) in preterm infants with small gestational age.^38^, ^39^, ^40^ Preterm infants born less than 27 weeks of gestation showed growth failure which contributes to the risk of BPD development. As a result of BPD, it can be observed in changes of lung pathophysiology of the affected patients. A summary of the affected lung pathophysiology can be seen in Figure 1.

## 3 MESENCHYMAL STEM CELLS AS A TREATMENT FOR BPD

Through an increase in stem cell research, scientists have established the therapeutic properties of stem cells, which are undifferentiated cells that possess the ability to differentiate into specialized cells. This contributes to their characteristics of self‐renewal, organogenesis, maintenance, repair and tissue regeneration.^41^ For instance, MSCs, a class of adult stem cells obtained from mesoderm, exhibit the benefits of easy isolation and expansion in addition to displaying multipotency and pleiotropy in several injury models due to which they are highly effective in stem cell biology. Their incorporation in the treatment of neonatal lung injuries has showcased the capability of MSCs to protect lungs and implement paracrine‐mediated activity for therapeutic benefits such as decreased immunogenicity and non‐tumourigenicity.^42^, ^43^ Human umbilical cord‐derived mesenchymal stem cells (hUC‐MSCs) are easily acquired, highly proliferative and exhibit an immensely high paracrine action when compared with other sources. Their therapeutic activity is proven through their ability to reduce lung inflammation, fibrosis, angiogenesis and apoptosis in several animal pulmonary disease models displaying pulmonary fibrosis and acute lung injuries, which include BPD.^44^, ^45^, ^46^

The idea of incorporating the regenerative properties of MSCs as a possible treatment strategy for BPD was put into motion with the first in‐human clinical trial submitted by Medipost Co. Ltd. in December 2010 in South Korea (NCT01297205).^47^ Their success in proving the safety and feasibility of stem cell incorporation for BPD led to the introduction of PNEUMOSTEM®, a product of allogeneic sources of stem cells derived from human umbilical cord blood (hUCB‐MSCs). As of late, the clinical trials concerning PNEUMOSTEM® have reached phase II clinical trials with the trials currently ongoing in South Korea along with its passing phase II in the United States. Due to its excellent potential and milestones unachievable by other stem cell products, it has been designated as an Orphan Drug by the US FDA and EMA for the trial in South Korea in addition to being granted a Fast‐Track Designation from the US FDA.^48^ That same trial then became the standard for all the clinical trials that followed in investigating the use of stem cells for BPD.

As of late, there exists multiple in‐human clinical trials concerning the use of stem cells for BPD with most of them, if not all, managing to prove their safety and feasibility with more products under development. Notable products under development include UNEX‐42, first introduced by United Therapeutics. The product is a relatively new approach to stem cell therapy for BPD as it is prepared from the extraction of extracellular vesicles from allogeneic sources of stem cells obtained from the bone marrow. Currently, UNEX‐42 is still under an ongoing trial situated in phase I and is estimated to be completed by the end of 2021 (NCT03857841).^49^ Another product to look forward to will be Meridigen's product, labelled as UMC119‐01. The source of UMC119‐01 are stem cells derived from the umbilical cords of neonates. This study is currently still in the recruiting phase and is expected to be completed nearing the end of 2022 (NCT03631420).^50^ The list of clinical trials regardless of its completion status is as listed in Table 2.^14^, ^47^, ^49^, ^50^, ^51^, ^52^, ^53^, ^54^, ^55^, ^56^, ^57^, ^58^, ^59^, ^60^, ^61^, ^62^, ^63^, ^64^, ^65^, ^66^, ^67^, ^68^

**TABLE 2:** Summary of active/completed phase I and II clinical trials using MSCs to treat patients with BPD

Study	NCT number	Cell type	Route	Number of patients (*n*)	Dosage (million cells/kg)
**MSC drug on‐going clinical trials**					
Medipost Co Ltd.^51^, ^52^	NCT02023788	PNEUMOSTEM	Intratracheal	8	10–20
	NCT04003857		Intratracheal	60	10
United Therapeutics^49^	NCT03857841	UNEX‐42	Not specified	18	20–200 pmol phospholipid/kg
Meridigen Biotech Co., Ltd.^50^	NCT03631420	UMC119‐01	Not specified	9	3–30
**Phase II**					
Medipost America Inc.^53^	NCT02381366	hUCB‐MSCs	Intratracheal	12	10–20
Children's Hospital of Fudan University^52^	NCT03645525	hUC‐MSCs	Intratracheal	180	20
Children's Hospital of Chongqing Medical University^54^	NCT03774537	hUC‐MSCs	Intravenous	20	1–5
Children's Hospital of Chongqing Medical University^55^	NCT03601416	hUC‐MSCs	Intravenous	72	2.5–5
Medipost Co. Ltd^56^	NCT03392467	hUC‐MSCs	Intraperitoneal	60	Not specified
**Phase I**					
Medipost Co. Ltd^47^	NCT01297205	hUCB‐MSCs	Intratracheal	9	10–20
Samsung Medical Center^57^	NCT01632475	hUCB‐MSCs	Intratracheal	9	10–20
Children's Hospital of Chongqing Medical University^58^	NCT03558334	hUC‐MSCs	Intravenous	12	1–5
Fundacion para la Investigacion Biomedica del Hospital Universitario Ramon y Caja^59^	NCT02443961	hUC‐MSCs	Not specified	10	5
Daping Hospital and the Research Institute of Surgery of the Third Military Medical University^60^	NCT03378063	hUCB‐MSCs	Not specified	100	Not Specified
Children's Hospital of Chongqing Medical University^61^	NCT03873506	hUC‐MSCs	Intravenous	30	1–5
Guangdong Women and Children Hospital^62^	NCT03683953	MSCs	Intratracheal	200	25
China Medical University Hospital^63^	NCT01207869	UC‐MSCs	Intratracheal	10	3
Ottawa Hospital Research Institute^64^	NCT04255147	Allogeneic UC‐MSCs	Intravenous	9	1–10
Vinmec Research Institute of Stem Cell and Gene Technology^65^	NCT04062136	hUC‐MSCs	Endotracheal and Intravenous	10	1
Liem et al.^66^	‐	Autologous BM‐MNCs	Intravenous	1	1
Lim et al.^67^	‐	Allogeneic hAECs	Intravenous	6	1
Baker et al.^68^	‐	hAECs	Intravenous	24	2–30
Nguyen et al.^14^	‐	Allogeneic UC‐MSCs	Intravenous	4	1

## 4 MECHANISM OF ACTION AGAINST BPD

**FIGURE 2:**
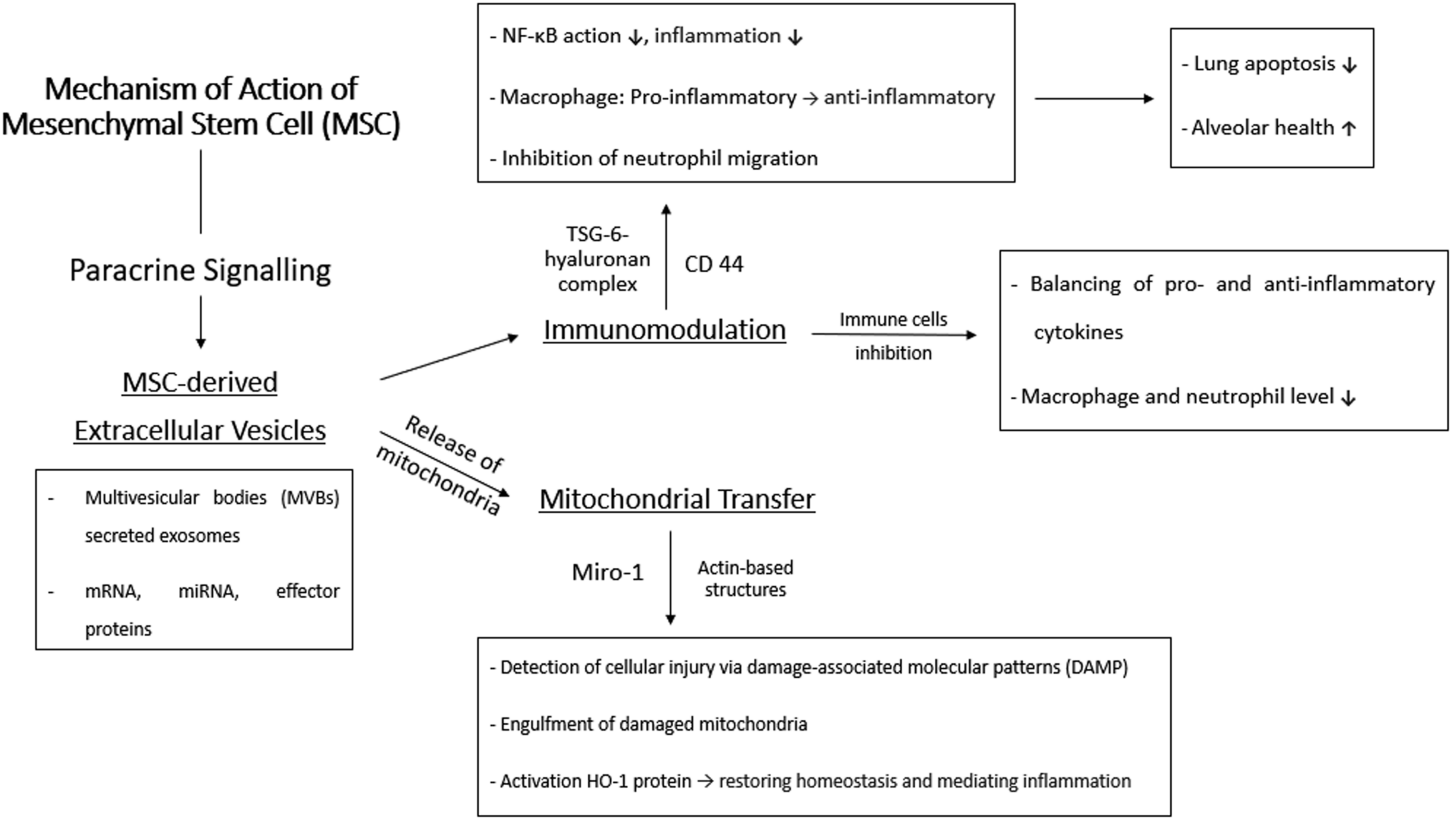
Mechanism of action of MSCs towards BPD in terms of the action of differentiated BPD and paracrine signalling of MSCs

What makes MSCs a highly sought‐after treatment plan for BPD lies in their hypothesized means of action that explains their therapeutic capabilities. Contrary to the initial hypothesized belief that the innate regenerative and differential capabilities of MSCs is responsible in ameliorating BPD, the idea that there is another mechanism besides the engraftment of MSCs was ascertained. Chang et al.^69^ could not form a direct connection between the mechanism of repair and regeneration of MSCs against lung injury. The idea that there is no need for the MSCs itself was reported where MSC‐conditioned media (MSC‐CM) presented a profound effect in preventing alveolar injury, preserving normal lung function and stimulating bronchoalveolar stem cells (BASCs) when compared with the introduction of bone marrow stem cells (BMSCs) themselves.^70^, ^71^, ^72^ In vivo studies both showed very low levels when introduced in BPD models that suggests that the engraftment of MSCs is not suitable as the primary mechanism in terms of lung protection.^73^, ^74^ Hence, for BPD, the main mechanism is hypothesized to be through paracrine signalling involving the release of specific soluble factors which triggers a cascade of events that can result in immunomodulation, antioxidation, anti‐inflammation and the release of signalling peptides. A summary of the mechanism of action of MSCs towards BPD is as shown in Figure 2.

### 4.1 Paracrine signalling

#### 4.1.1 Extracellular vesicles (exosomes)

Nanometer‐size extracellular vesicles/microvesicles known as exosomes are important mediators of intercellular signalling by the constituents, they carry which can vary between mRNA, mi‐RNA and effector proteins, involved in altering gene expressions.^75^, ^76^, ^77^ MSC‐derived exosomes follow the exocytosis pathway where they are secreted by multivesicular bodies (MVBs) when fused with the plasma membrane.^78^ The release of the exosomes allows for a change in biological processes mediated by their wide range of properties ranging from their regenerative ability to their immunomodulatory and disruptive properties with regards to the pathogenesis of various diseases including inflammatory diseases.^76^ Following the fusion, the exosomes are released to interact with target cells in the micro‐environment or transported to targeted regions through biological fluids. In vivo studies against BPD in animal models showed the effectiveness of MSC‐derived exosomes in mediating pulmonary hypertension arising from BPD and the disease as an overall and an increase in lung compliance as a whole when compared with MSCs without the presence of exosomes.^13^, ^79^ Dong et al.^80^ studied the relationship between miRNAs and the incidence of BPD where a strong correlation was found, suggesting that the exosome release mediated by MSCs could play a vital role in reversing BPD by altering/silencing gene expressions accordingly. Furthermore, Cho et al.^81^ found that MSC exosomes can decrease the eosinophil level, number of IgE and CD86+ cells in atopic dermatitis model that mimics the BPD environment.

Research centring around MSC‐derived exosomes is important in understanding its mechanism as a therapeutic treatment method for BPD, considering the positive results seen in BPD models. The role of MSC‐derived exosomes and its exact mechanism is yet to be elucidated; however, its role in facilitating mitochondrial transfer together with its potential immunomodulatory function is heavily endorsed. Willis et al.^82^ suggested a possibility where MSC‐derived exosomes can exert their anti‐inflammatory properties in BPD models by reprogramming the recipient's immune system. More specifically, altering the phenotype of existing pulmonary macrophages through a shift in the M1/M2 ratio present in macrophages which are the pro and anti‐inflammatory markers, respectively. The mitochondria constituent of exosomes is expected to be transferred to resident pulmonary cells including macrophages. Additionally, its immunomodulatory property is made possible due to the presence of an anti‐inflammatory protein, TNF‐stimulated gene 6 protein (TSG‐6) in MSCs.^83^ Both mechanisms interconnected with the release of exosome bodies will be elaborated upon in the following sections.

### 4.2 Immunomodulation

MSCs have been found to be capable of interacting with both the innate and adaptive immune system, successfully lowering the immunogenicity and managing to go around it in a way as to not provoke an immune response.^76^ TSG‐6, an inflammatory exosome protein, is composed of link modules, enabling its binding to notable glycosaminoglycan, including that of hyaluronan and chondroitin sulphate.^84^ Hyaluronan is involved in processes surrounding inflammation where it acts to stabilize the extracellular matrix, maintaining structural and functional integrity.^85^ The bound TSG‐6‐hyaluronan complex can interact with CD44 receptor, a cell surface glycoprotein present on macrophages. Once the interaction is successful, one of the three main inflammatory pathways, the action of the NF‐κB pathway is then reduced where inflammation is subdued. Moreover, Mittal et al.^86^ published findings wherein TSG‐6 promotes the conversion of macrophages from pro‐inflammatory to an anti‐inflammatory composition by suppressing pro‐inflammatory transcription factors. Furthermore, neutrophil migration to the inflammation site is also inhibited. Bryan et al.^87^ and Chaubey et al.^88^ reported a decrease in lung apoptosis and a major improvement in alveolar disruption in BPD models treated with MSCs where high levels of TSG‐6 were present in the lung tissues, supporting the theory concerning the role of TSG‐6.

MSCs are also able to inhibit the action of immune cells, B cells, dendritic cells and natural killer cells by suppressing their proliferative activity or by stimulating the expression of certain immune cells. BPD affects the resident pulmonary immune cells possibly because of an imbalance in the levels of pro and anti‐inflammatory cytokines.^89^ These events are orchestrated by the release of immunomodulatory factors who are important pulmonary biomarkers of BPD‐ transforming growth factor‐β1 (TGF‐β1), IL‐6, IL‐10, IL‐16 and so forth.^89^ What ensues is a rise in macrophages and neutrophil levels seen in infants with BPD complications to repair and restore the damage inflicted on the lungs.^89^ The addition of MSCs are expected to balance up the levels of both pro and anti‐inflammatory cytokines by decreasing and increasing the levels accordingly.^90^ Immunomodulatory functions of MSCs against BPD are proposed as the main mechanism behind MSCs where reduction in macrophage and neutrophil levels were observed upon MSC administration in vitro with evidence wherein MSC‐CM prevented the proliferation of IL‐1α and TNF‐α, both pro‐inflammatory cytokines resulting from activated macrophages.^91^ Aslam et al.^70^ supported the theory by enlisting murine models with attenuated lung injury. As a result, tissue repair was observed, and an improvement was seen in lung morphology.^70^ Similarly, MSC transplants in murine models with acute lung injury and bleomycin‐induced lung injury and fibrosis showed a decrease in macrophage protein levels and an increase in releasing cytokines (IL‐1ra) that can compete with pro‐inflammatory cytokines (IL‐1b).^92^, ^93^ The immunomodulatory function of MSCs exosomes was also found to greatly affect pulmonary macrophages by mediating inflammation accompanying induced hyperoxia in animal models.^94^

### 4.3 Mitochondrial transfer

There has been evidence suggesting the efficacy of cell‐to‐cell contact involving mitochondrial transfer from MSCs to the damaged cells to fill up for their inadequate respiratory function. The likelihood of mitochondrial transfer by TNT in bronchial epithelial cells was suggested through in vitro experiments.^95^, ^96^ Protective outcomes with regards to mitochondrial transfer were seen in treating organ injury (lung, kidney, spinal cord etc.).^97^ MSC‐mediated mitochondrial transfer functions to effectively mitigate any damage inflicted on the mitochondria and the bronchopulmonary system altogether, because of a plethora of reactive oxygen species (ROS) production bringing about oxidative stress onto the cells. Mitochondrial homeostasis is never achieved, leading to a damaged mitochondrion, like in the case of BPD.^98^ Various pathways have been identified which could possibly account for the transfer of mitochondria via actin‐based structures such as tunnelling nanotubes (TNT), gap junctions, micro‐vesicles and cellular fusion.^99^ The navigation of mitochondria to the site of cell injury is mainly mediated by Mitochondrial Rho GTPase 1 (Miro‐1), an adaptor protein known to be involved in mitochondrial intercellular transport.^100^

Miro‐1 is part of a protein complex together with Myo 10, Myo 19, TRAK 1, TRAK 2 and KLF 5, which acts in regulating the transport mechanism of the mitochondria and subsequently allowing its movement through the cytoplasmic nanotubes.^100^, ^101^, ^102^ The importance of Miro‐1 is confirmed in multiple studies where its expression enhances MSCs mitochondrial transfer efficiency and vice versa.^103^, ^104^, ^105^ The hypothesized mechanism in which MSCs can detect the respiratory damage brought upon by a damaged mitochondria lies in environmental cues released by the injured cells.^101^, ^106^ These are in the form of the damaged mitochondria itself and its associated products (mtDNA, *N*‐formyl peptides and lipids) where they play a vital role in inflammatory responses and are recognized by surrounding cells as damage‐associated molecular patterns (DAMPs).^107^ Mahrouf‐Yorgov et al.^106^ were able to show that the mitochondrial transfer is initiated when MSCs meet the damaged mitochondria, leading to engulfment of the latter. The chain reaction that follows leads to the activation of the HO‐1 protein responsible for mediating vascular inflammation and restoring mitochondrial homeostasis and overall cell state.^108^ ROS production has also been postulated as a trigger in the release of mitochondrial MSCs.^106^, ^109^

Mitochondrial transfer has shown its bioenergetic potential in lung conditions impacted by oxidative stress which is significant in the pathogenesis of BPD. The delivered mitochondria are able to conform to the energy requirement of cells by differentiating from its dormant state into an active state accordingly.^101^ This results in an increase in DNA/RNA production, positively affecting the number of respiratory enzymes along with oxygen consumption levels and preserving ATP/respiratory levels.^101^ Following alveolar/airway barrier disruption, a partial restoration in dysfunctional mitochondria accompanied by usual/normal oxidative phosphorylation levels, activated antioxidant pathways, and an improvement in lung health was observed when MSC mitochondria were present as compared with the introduction of MSCs in the absence of mitochondria.^96^, ^97^, ^110^ Islam et al.^109^ demonstrated its significance by introducing BM‐MSCs in vivo in mice models with induced acute lung injury. As a result, mitochondrial transfer took place through nanotubes and micro‐vesicles where the restoration of mitochondrial bioenergetics in the lungs was observed. Another condition like the pathogenesis of BPD, chronic obstructive pulmonary disorder (COPD), was also experimented on where mitochondrial transfer took place, passing through TNT, reaching the lung epithelium tissues where alveolar damage was successfully restored.^95^ It was also suggested that mitochondrial transfer might possess anti‐inflammatory potential, a key side effect of individuals suffering from BPD.^95^

## 5 BARRIERS IN MSC THERAPY

**FIGURE 3:**
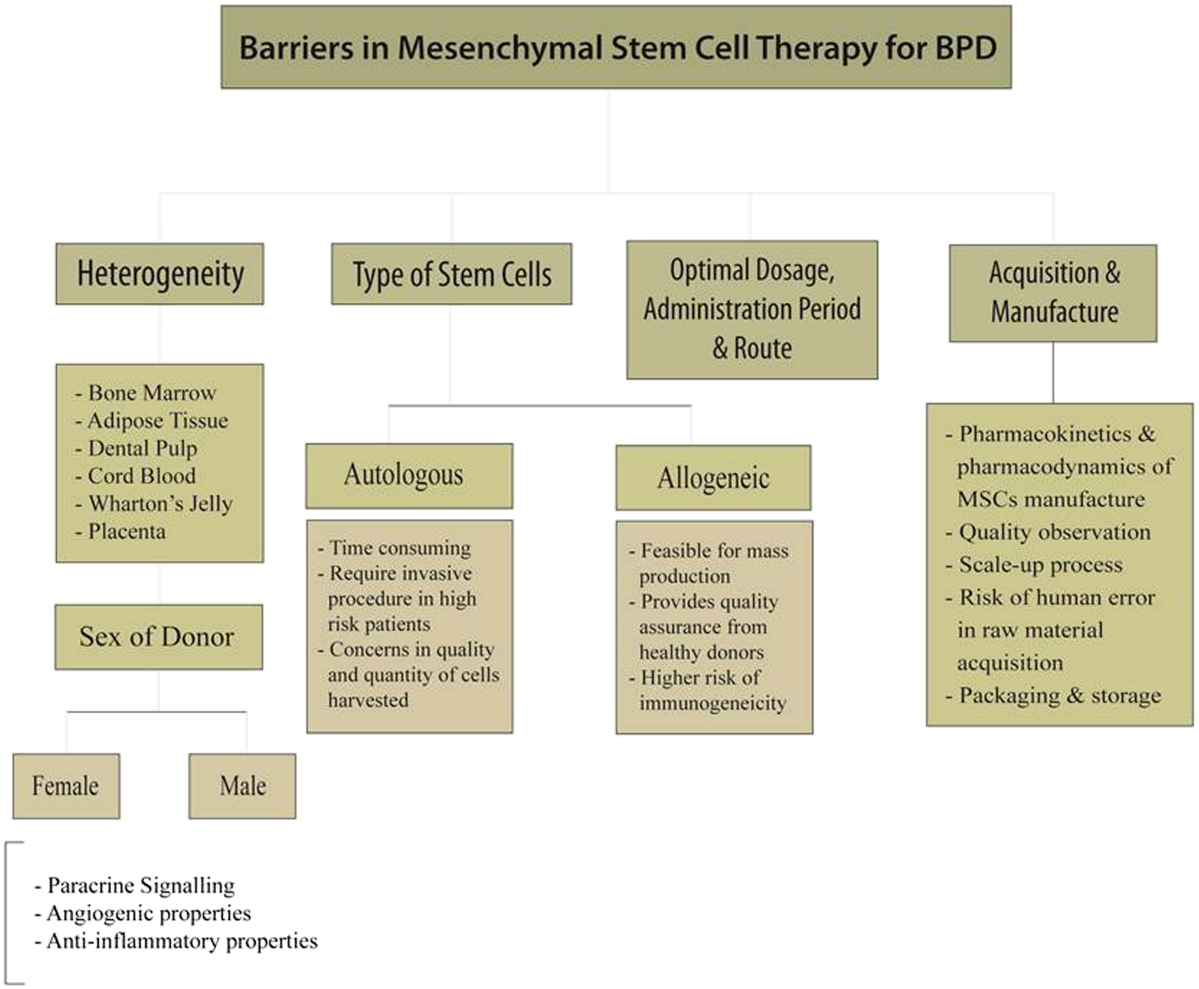
Barriers and obstacles faced in development of MSC therapy as a treatment for BPD

Although trials incorporating MSC for BPD are limited, provided only a handful of clinical trials are completed with the rest currently ongoing, the statistics remain favourable towards the introduction of MSC as a permanent treatment strategy both in terms of safety as well as efficacy. However, with great success comes ever growing concerns that have to be addressed before stem cell products like PNEUMOSTEM®, UNEX‐42 and UMC119‐01 are ready to be marketed. A summary of the barriers and obstacles faced in developing MSC therapy can be found in Figure 3.

### 5.1 Allogeneic vs autologous sources

Yang et al.^111^ assessed the safety of incorporating autologous hUCB in preterm infants (less than 37 weeks gestational age) for treatment of diseases including that of BPD. The treatment was considered a success where favourable results were obtained as no complications arose, nor were there any reported adverse events. This study proves the usage of autologous sources of MSCs for BPD; however it does not diminish the fact that there are a good deal of factors and questions to be taken into consideration. Will the amount of stem cells harvested be sufficient? Are the stem cells harvested healthy? Do they still maintain their regenerative and protective capabilities? Every individual is indeed different—their state of well‐being, the amount of stem cells they have, their stem cell capability—which increases complexities should only autologous therapies be carried out. This explains why most clinical trials on stem cell therapy pertaining to BPD involved the use of allogeneic sources of MSCs rather than autologous sources. Allogeneic sources are not only more feasible and suitable for mass production to cater for a larger scale of research but they are also efficient in handling the concerns that autologous therapy poses. To ensure the stem cells harvested for allogeneic therapies do not carry the above concerns on its state and capacity, they can be harvested from healthy donors.^112^ It provides quality assurance and control on the stem cells being used together with the fact that it is less time consuming and cheaper considering the stem cells harvested can be used for administration within multiple BPD patients. The only downside to allogeneic therapy would be that they possess a considerably higher risk of graft‐versus‐host disease (GVHD) wherein the immune system is unable to recognize the cells and targets them as foreign, leading to the inevitable rejection of the allogeneic cells which is not a problem in autologous therapies.^113^ Implementation of cell‐free supernatants is an approach that may overcome issues related to allogeneic therapy as results concluded from animal trials indicate the efficiency of MSC‐based treatments is moderated, in BPD, mainly by paracrine activity.^114^

### 5.2 Heterogeneity

Heterogeneity of stem cells is attributed to the many different areas they can be harvested from. To name a few are bone marrow, adipose tissue, dental pulp, cord blood, Wharton jelly and the placenta.^115^, ^116^, ^117^ The stem cells harvested differ in their characteristics. For instance, umbilical cord derived stem cells (hUCB‐MSCs, hUC‐MSCs and hAEC‐MSCs) are preferable as compared with bone marrow derived stem cells as they are found to be easier to harvest coupled with advanced characteristics such as having a higher proliferative capacity, lower antigenic properties and a stronger paracrine effect.^43^, ^44^ Contributions to the heterogeneity of MSCs lies in the sex of the donor. Sammour et al.^118^ reported that MSCs harvested from female donors displayed stronger paracrine effects with greater angiogenic and anti‐inflammatory properties which resulted in an overall reduction in neonatal hyperoxia‐induced lung diseases which are inclusive of BPD. The mechanism of action of MSCs against BPD relies heavily on its paracrine signalling. With that being so, there is no need for the MSC itself and an MSC conditioned media would suffice. This in itself causes variability when selecting the cell population with the most abundant humoral factors to be used and the same problem follows for exosomes. Looking at the pathophysiology of BPD, it is evident that it is a multifactorial disease, which requires a more precise selection on the MSC to be used based on its heterogeneity.^119^ Therefore, the emphasis on the need of a standardized isolation protocol that have been proven to be the most advantageous, fully utilizing the protective and regenerative effects of MSCs for BPD is called for.

### 5.3 Acquisition and manufacturing

The acquisition of raw materials for stem cell therapies is a complex process requiring aseptic processing protocols. Moreover, their mechanism within the body differs from medicinal drugs which makes the pharmacokinetics and pharmacodynamics challenging.^120^ To market stem cells as an ‘off‐the‐shelf’ medication for BPD, easily accessible in large bulks, large‐scale manufacturing is a definite process. This whole process of scaling up involves a long and straining cycle from the collection, storage, process and cryopreservation of stem cells to the subjection of stem cells for further improvisation, formulating it to be suitable in accordance with the targeted therapies.^120^ Through analysis of suppliers and their rate of purity and sterilization techniques, high quality raw materials required for an uninterrupted and licensed medicinal product for advanced clinical trials can be secured following the success of early clinical phases. Moreover, to prevent contamination, raw materials can be aliquoted into desired vials and packaged immediately at the supplier firm. With the balance of standardization and customization in addition to introducing unique raw material formats, that is, instigation of kits, combining cytokines with media, and reconstitution cytokines, barriers adhering to timeliness and raw material stability can be overcome. Moreover, with standardization of products, the cost, manufacturing time and number of suppliers will be lowered in comparison with customized therapies. This technique also supports a larger number of BPD patients, accessibility, feasibility due to the reduced cost of manufacturing and ‘off‐the‐shelf’ medication. Personalized equipment designed in collaboration with the suppliers can also ease the process of manufacturing.

The original state of stem cells following subculturing gets compromised and the number of times where the cells are subjected to the cell culture cycle impacts the capacity of the stem cells as it is known that they are more susceptible to an impairment in immunomodulatory property as well as experiencing senescence. The prospect of genomic instability is also present from continuous expansion of the MSCs. Hence, the usage of MSCs is restricted to cells that are in the earlier subculturing stages to observe the full benefits of MSCs during BPD treatment. However, that statement is not fully validated as although there have been indications in which cryopreservation encourages functional impairment of the cells, the data gathered is also limited which prompts for more extensive research regarding this area.^121^ Researchers have observed and elucidated the genetic stability of hUC‐MSCs expanded in vitro in cultures up to passage P13.^122^ However, the possibility of carcinogenic transformation of transplanted pluripotent cells is another risk factor that does not have a definitive solution.^123^ As karyotype analyses do not account for copy number variants (CNV) or mutations, the incorporation of array‐comparative genomic hybridization (aCGH) at later passages prior to stem cell transplantation, quality control and long term follow ups for adverse events in neonates with BPD can be induced to ensure genetic stability.

### 5.4 Optimal dosage, administration period and route of administration

Undefined factors such as the optimal dosage, administration period and route are important factors that impact the effect of MSCs. Chang et al.^124^ demonstrated the efficacy of hUCB‐MSCs on rodents with hyperoxia lung injuries and suggested the time‐dependent nature of administration, where administration at an earlier stage could better the efficacy of the MSCs against BPD as compared with administration at a later stage. When reviewing clinical trials that have been completed or are underway, the range of dosage given was significant (1–20 million cells/kg) and all doses were well tolerated in all patients which could suggest that the amount of MSCs given does not need to be restricted to a certain amount and as long as the amount administered is within the range provided by previous clinical trials, it is considered safe and effective. As for the number of doses to be administered, there is no conclusive data proving one option is more superior than the other, although multiple doses of MSC have shown greater efficacy in managing intraventricular haemorrhage, a condition highly associated with BPD.^125^ Besides that, all other factors listed remain unclear, including a standardized clinical protocol. To safely determine the effective dosage, including the number of doses needed and the best timeframe, more dose‐escalation studies will prove beneficial in future clinical trials.

One of the most debated issues concerning MSC therapy for BPD is the route of administration, mainly the intratracheal and the intravenous pathway. Both have shown their efficacy with varying effects, with more research focused on the former pathway. Efficacy when administered intratracheally appeared to be better than when it was administered intravenously, but its usage was only restricted to preterm who are severely affected with BPD.^114^ The explanation lies in the fact that preterm infants, in general, are put on ventilator support for only a short period which emphasizes the need to transition mainly into the intravenous pathway. The selection of preterm infants to receive the stem cells is also an important factor. The absence of a reliable biomarker poses a problem in understanding and observing the therapeutic efficacies of the treatment using MSCs. With the presence of a biomarker, the selection of preterm infants is more reliable with the selection of only preterm infants with high risk of BPD incidence. Thus, effects resulting from the MSC treatment on preterm infants can also be easily predicted with minimal issues. The findings of a biomarker can further aid in the use of allogeneic sources of MSCs by heavily monitoring the BPD biomarker.^114^

## 6 CONCLUSION AND FUTURE PROSPECTS

With the advancement of technology and research in regenerative medicine, the development of MSC therapy may be a breakthrough in treatment of BPD within preterm infants. The emergence of numerous clinical trials in the recent years on MSC products such as PNEUMOSTEM®, UNEX‐42 and UMC119‐01 with successful results has proved the safety and practicality of integrating stem cell therapy as one of the treatments for BPD. However, with the ongoing clinical trials, there remain barriers and hurdles faced in developing an off the shelf MSC medication for BPD in which the processes involved in the formulation of the MSC product have been covered up till the manufacturing area with the same concerns in mind, how do we present the product in such a way that it can be marketed as an ‘off‐the‐shelf’ type of medication or otherwise as prescription medication? One particular area to keep sight off would be the form of the product it is marketed in, in the future. With the current trials focused on administrations that require expertise and a certain level of knowledge, there are health and ethical concerns that should the product be marketed as a ‘self‐injection’ type of product, there is the assumption that the public is equipped with enough knowledge on how to self‐administer or administer it on the affected infants. The need for regulations on the usage and/or prescription of said MSC drug must be looked into, to avoid abuse or misuse of the product. Another way is to investigate other possible routes of administration that can dispel those concerns. One of them would be to investigate the oral route of administration in future clinical trials. Bearing the heterogeneity of the stem cells in mind, in addition to the processes leading to the marketing of the product, more research and investigation is required before there can be the possibility of an ‘off‐the‐shelf’ and a ‘one‐size‐fits‐all’ stem cell medication for BPD.

## CONFLICTS OF INTEREST

Kong‐Yong Then, Khong‐Lek Then and Soon‐Keng Cheong are directors of CryoCord Sdn Bhd and declare direct share interest in the company whereas all other authors declare no conflict of interest.

## ETHICS STATEMENT

Ethics approval was not required for this study.

## AUTHOR CONTRIBUTIONS

V.G. contributed to the conceptualization and writing (original draft, review and editing) of the manuscript. E.T., M.Z. and W.H.L., contributed to the writing (original draft and review) of the manuscript. K.Y.T., K.L.T., M.G. A.K.D. and S.K.C. contributed to the writing (review and editing).

## Data Availability

Data sharing not applicable to this article as no datasets were generated or analysed during the current study.
